# Supplementary material for: Magnetism and Grain-Size Distribution of Particles Deposited on the Surface of Urban Trees in Lanzhou City, Northwestern China
Source: Int J Environ Res Public Health. 2021 Nov 14;18(22):11964. doi: 10.3390/ijerph182211964 (PMC8622433; doi:10.3390/ijerph182211964)
Supplement: Supplementary file 1 [file ijerph-18-11964-s001.zip › ijerph-1380508-supplementary.pdf]

Table S1. Descriptive statistics for SIRM, PM weight and grain-size distribution of particles deposited on the surface of needles

Samples	SIRM	Weight	Particle size			
	$\times 10^{-5} Am^2 \cdot kg^{-1}$	$g \cdot kg^{-1}$	0.02-1 μm/%	1-2.5 μm /%	2.5-10 μm /%	>10μm /%
						All sample(n=480)
min	2.08	0.56	2.33	3.26	16.13	50.07
max	659.49	67.69	6.44	8.26	37.44	77.88
mean	89.37	14.34	4.19	6.09	27.17	62.54
s.d	68.89	8.17	0.66	0.80	2.99	3.81
						T0 (height:120-600 cm)
min	25.92	4.62	2.73	3.26	20.14	57.10
max	659.49	67.69	5.00	7.48	33.29	73.79
mean	142.50	16.87	3.67	5.47	27.58	63.27
s.d	118.07	11.66	0.54	0.70	3.09	3.65
						T1 (height:100-540 cm)
min	27.61	4.04	2.84	3.95	21.25	50.07
max	314.46	49.25	6.44	8.17	37.44	71.96
mean	92.67	14.94	4.56	6.60	28.84	60.01
s.d	54.96	8.81	0.68	0.74	2.35	3.30
						T2 (height:100-560 cm)
min	29.12	7.09	2.71	4.16	17.03	54.50
max	183.77	37.77	6.10	8.26	32.01	75.65
mean	86.62	16.03	4.42	6.28	26.07	63.22
s.d	28.98	5.50	0.59	0.71	2.71	3.52
						T3 (height:100-560 cm)
min	6.63	3.44	2.55	4.17	17.77	56.61
max	158.38	35.49	5.16	7.31	32.81	74.79
mean	68.68	12.68	4.16	6.00	26.03	63.81
s.d	27.66	5.95	0.55	0.67	2.96	3.68
						T4 (height:100-560 cm)
min	2.08	0.56	2.33	3.66	16.13	57.05
max	197.54	40.32	5.14	7.30	32.76	77.88
mean	55.76	11.36	4.19	6.12	27.36	62.33
s.d	32.69	6.33	0.60	0.69	2.82	3.64

Table S2. results of one-way ANOVA performed of every four sampling directions (NE, SW, NW and SE) at sites T0-T4. Statistically significant differences (p<0.05) are highlighted in bold.

dependent variable	(I)direction	(J)direction	T0	T1	T2	T3	T4
SIRM	NE	NW	0.183	**0.060**	0.501	0.991	**<0.0001**
		SE	0.022	**<0.0001**	**<0.0001**	**0.001**	**0.227**
		SW	0.001	**<0.0001**	**<0.0001**	**0.516**	**<0.0001**
	NW	NW	0.183	0.060	0.501	0.991	**<0.0001**
		SE	0.322	**0.001**	**0.002**	**0.001**	**0.004**
		SW	0.050	**<0.0001**	**<0.0001**	0.509	0.259
	SE	NE	**0.022**	**<0.0001**	**<0.0001**	**0.001**	0.227
		NW	0.322	**0.001**	**0.002**	**0.001**	**0.004**
		SW	0.324	0.435	0.690	**0.007**	**<0.0001**
	SW	NE	**0.001**	**<0.0001**	**<0.0001**	0.516	**<0.0001**
		NW	0.050	**<0.0001**	**<0.0001**	0.509	0.259
		SE	0.324	0.435	0.690	**0.007**	**<0.0001**
weight	NE	NW	0.506	**0.044**	0.914	0.340	**0.073**
		SE	0.648	**<0.0001**	**0.015**	0.068	0.476
		SW	0.222	**<0.0001**	**0.002**	0.856	**0.016**
	NW	NW	0.506	**0.044**	0.914	0.340	**0.073**
		SE	0.263	**0.007**	**0.020**	**0.006**	0.275
		SW	**0.061**	**0.028**	**0.003**	0.257	0.529
	SE	NE	0.648	**<0.0001**	**0.015**	0.068	0.476
		NW	0.263	**0.007**	**0.020**	**0.006**	0.275
		SW	0.442	0.558	0.527	0.099	0.087
	SW	NE	0.222	**<0.0001**	**0.002**	0.856	**0.016**
		NW	**0.061**	**0.028**	**0.003**	0.257	0.529
		SE	0.442	0.558	0.527	**0.099**	0.087
0.02-1 μm	NE	NW	0.199	**0.003**	**0.008**	0.819	0.782
		SE	0.443	**0.013**	0.762	0.624	0.926
		SW	0.219	**0.027**	0.274	0.818	0.748
	NW	NW	0.199	**0.003**	**0.008**	0.819	0.782
		SE	0.590	**<0.0001**	**0.003**	0.467	0.703
		SW	0.957	**<0.0001**	0.109	1.000	0.540
	SE	NE	0.443	**0.013**	0.762	0.624	0.926
		NW	0.590	**<0.0001**	**0.003**	0.467	0.703
		SW	0.628	0.690	0.164	0.463	0.812
	SW	NE	0.219	**0.027**	0.274	0.818	0.748
		NW	0.957	**<0.0001**	**0.109**	1.000	0.540
		SE	0.628	0.690	0.164	0.463	0.812
1-2.5 μm	NE	NW	0.065	**0.001**	**0.042**	0.975	0.362
		SE	**0.003**	**0.038**	0.393	0.707	0.564

		SW	**0.004**	0.098	0.927	0.195	0.678
	NW	NW	0.065	**0.001**	0.042	0.975	0.362
		SE	0.240	**<0.0001**	**0.004**	0.680	0.723
		SW	0.292	**<0.0001**	0.051	0.200	0.176
	SE	NE	**0.003**	**0.038**	0.393	0.707	0.564
		NW	0.240	**<0.0001**	**0.004**	0.680	0.723
		SW	0.911	0.607	0.345	0.091	0.308
	SW	NE	**0.004**	0.098	0.927	0.195	0.678
		NW	0.292	**<0.0001**	0.051	0.200	0.176
		SE	0.911	0.607	0.345	0.091	0.308
2.5-10 µm	NE	NW	0.140	**0.020**	0.732	**0.026**	**0.041**
		SE	**0.026**	0.367	**0.002**	**0.035**	**0.025**
		SW	0.349	0.426	0.090	0.422	0.068
	NW	NW	0.140	**0.020**	0.732	**0.026**	**0.041**
		SE	**<0.0001**	0.187	**0.005**	0.901	0.852
		SW	0.582	0.124	0.180	0.139	0.816
	SE	NE	**0.026**	0.367	**0.002**	**0.035**	**0.025**
		NW	**<0.0001**	0.187	**0.005**	0.901	0.852
		SW	**0.002**	0.884	0.127	0.175	0.673
	SW	NE	0.349	0.426	0.090	0.422	0.068
		NW	0.582	0.124	0.180	0.139	0.816
		SE	**0.002**	0.884	0.127	0.175	0.673
>10 µm	NE	NW	0.881	**0.003**	0.539	0.056	0.120
		SE	**0.003**	0.952	0.008	0.068	0.100
		SW	0.582	1.000	0.268	0.701	0.234
	NW	NW	0.881	**0.003**	0.539	0.056	0.120
		SE	**0.002**	**0.004**	**0.001**	0.928	0.936
		SW	0.479	**0.003**	0.089	0.115	0.704
	SE	NE	**0.003**	0.952	**0.008**	0.068	0.100
		NW	**0.002**	**0.004**	**0.001**	0.928	0.936
		SW	**0.014**	0.952	0.109	0.137	0.642
	SW	NE	0.582	1.000	0.268	0.701	0.234
		NW	0.479	0.003	0.089	0.115	0.704
		SE	**0.014**	0.952	0.109	0.137	0.642

Table S3. Results of one-way ANOVA performed of every three sampling height ranges(L, M and H) at sites T0-T4. Statistically significant differences ($p<0.05$) are highlighted in bold.

dependent variable	(I)height range	(J)height range	T0	T1	T2	T3	T4
SIRM	L	M	**0.019**	**0.003**	0.970	0.059	0.722
		H	**<0.0001**	**<0.0001**	0.974	0.053	0.854
	M	L	**0.019**	**0.003**	0.970	0.059	0.722
		H	**0.010**	**0.043**	0.992	0.948	0.516
	H	L	**<0.0001**	**<0.0001**	0.974	0.053	0.854
		M	**0.010**	**0.043**	0.992	0.948	0.516
weight	L	M	**<0.0001**	**<0.0001**	0.544	0.117	0.257
		H	**<0.0001**	**<0.0001**	0.407	0.550	0.544
	M	L	**<0.0001**	**<0.0001**	0.544	0.117	0.257
		H	**0.001**	**<0.0001**	0.826	0.217	0.491
	H	L	**<0.0001**	**<0.0001**	0.407	0.550	0.544
		M	**0.001**	**<0.0001**	0.826	0.217	0.491
0.02-1 μm	L	M	0.813	0.357	0.356	0.096	**0.045**
		H	**0.023**	**0.093**	0.378	**0.001**	**0.011**
	M	L	0.813	0.357	0.356	0.096	**0.045**
		H	**0.002**	0.401	0.917	0.081	0.618
	H	L	**0.023**	0.093	0.378	**0.001**	**0.011**
		M	**0.002**	0.401	0.917	0.081	0.618
1-2.5 μm	L	M	0.635	**0.031**	0.837	**0.016**	0.054
		H	0.061	**0.017**	0.153	**<0.0001**	**0.027**
	M	L	0.635	**0.031**	0.837	**0.016**	0.054
		H	**0.003**	0.852	0.058	0.057	0.834
	H	L	0.061	**0.017**	0.153	**<0.0001**	**0.027**
		M	**0.003**	0.852	0.058	0.057	0.834
2.5-10 μm	L	M	0.282	**0.001**	**0.005**	**0.013**	0.840
		H	**0.023**	**<0.0001**	**<0.0001**	**<0.0001**	0.171
	M	L	0.282	**0.001**	**0.005**	**0.013**	0.840
		H	0.146	**<0.0001**	**0.001**	**0.047**	0.173
	H	L	**0.023**	**<0.0001**	**<0.0001**	**<0.0001**	0.171
		M	0.146	**<0.0001**	**0.001**	**0.047**	0.173
>10 μm	L	M	0.448	**0.003**	0.055	**0.002**	0.881
		H	**0.004**	**<0.0001**	**<0.0001**	**<0.0001**	0.571
	M	L	0.448	**0.003**	0.055	**0.002**	0.881
		H	**0.009**	**0.012**	**0.004**	**0.040**	0.392
	H	L	**0.004**	**<0.0001**	**<0.0001**	**<0.0001**	0.571
		M	**0.009**	**0.012**	**0.004**	**0.040**	0.392
